# Feasibility of weight loss in obese atrial fibrillation patients attending a specialist arrhythmia clinic and its impact on ablation outcomes

**DOI:** 10.1002/joa3.12432

**Published:** 2020-09-13

**Authors:** Wern Yew Ding, Nikola Kozhuharov, Shui Hao Chin, Matthew Shaw, Richard Snowdon, Gregory Y. H. Lip, Dhiraj Gupta

**Affiliations:** ^1^ Liverpool Heart and Chest Hospital Liverpool Centre for Cardiovascular Science University of Liverpool United Kingdom; ^2^ Liverpool Heart and Chest Hospital Liverpool United Kingdom; ^3^ Cardiovascular Research Institute Basel (CRIB) Department of Cardiology University Hospital Basel Basel Switzerland; ^4^ Aalborg Thrombosis Research Unit Department of Clinical Medicine Aalborg University Aalborg Denmark

**Keywords:** ablation, atrial fibrillation, outcome, specialist clinic, weight loss

## Abstract

**Background:**

The feasibility of significant weight reduction in a specialist arrhythmia service, and its impact on atrial fibrillation (AF) ablation outcomes are unclear. We aimed to assess these factors in a real‐world cohort in the United Kingdom.

**Methods:**

Patients from one specialized arrhythmia clinic were instructed to follow the “Intermittent Fasting 5:2 diet” (“diet group”, n = 50), and their outcomes were compared to a propensity matched cohort who received no specific dietary advice (“control group”, n = 42). The primary outcome was recurrence of AF or atrial tachycardia (AT) at 12 months postablation, with or without drugs.

**Results:**

Body weight and body mass index (BMI) at baseline were 105.0 (±15.3) kgs and 36.0 (±4.0), respectively. Baseline characteristics between the two groups were comparable. Patients in diet group experienced a mean weight loss of 8.2 (±7.1) kgs prior to AF ablation (*P* < .01 for comparison to baseline and control group). About 14 (28%) patients in the diet group lost >10% of their body weight. Overall, 11 (22%) patients in the diet group and five (12%) in the control group had AF recurrence at 1 year, *P* = .21. AF recurrence was similar in patients with BMI ≥ 35 (15%) as compared to BMI < 35 (19%), *P* = .60. There was one procedural complication (pulmonary edema) in the diet group.

**Conclusion:**

It is feasible to achieve significant weight reduction in obese AF patients in a specialist arrhythmia clinic setting with unsupervised dietary advice. Low rates of procedural complications and excellent medium‐term success rates were observed in this traditionally challenging population. Additional improvements in outcomes were not demonstrable in patients who exhibited significant weight loss.

## Graphical abstract

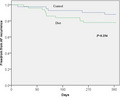

## 1 INTRODUCTION

Atrial fibrillation (AF) is a significant health condition that is associated with increased morbidity and mortality.^1^, ^2^, ^3^, ^4^ Obesity is recognized as an important risk factor for AF.^5^, ^6^ Obesity is associated with an increased left atrial size and decreased left ventricular diastolic function,^7^ which contribute to higher left atrial pressure.^8^, ^9^ These changes promote the initiation and maintenance of AF. Overall, obesity is related to worse outcomes in terms of AF symptom severity, frequency of AF‐related hospitalization, burden, and quality of life.^10^ Furthermore, obesity has adverse implications on the efficacy and safety of catheter AF ablation. Two independent meta‐analyses demonstrated that higher preablation body mass index (BMI) was linked to increased AF recurrence postablation.^11^, ^12^ Obese patients are also exposed to higher adverse events from AF ablation compared to nonobese patients.^13^, ^14^

Pathak and colleagues showed that targeted weight management in obese patients improved outcomes of AF ablation.^15^ On the other hand, Mohanty et al failed to demonstrate any benefits on ablation outcomes with weight reduction in patients with long‐standing persistent AF.^16^ As such, the data evaluating the role of weight reduction alone for improving the outcomes of catheter AF ablation are scarce and conflicting. Recently, the National Health Service (NHS) in the United Kingdom has proposed imposing severe restrictions to catheter ablation to obese AF patients, including the requirement to lose at least 10% body weight for them to be eligible (*circular 7th January 2020*). In the present study, we aimed to evaluate the feasibility of weight reduction in a real‐life specialist arrhythmia clinic in the United Kingdom, and its effects on results of catheter AF ablation.

## 2 METHODS

Consecutive obese patients with symptomatic AF, with a BMI of greater than 30, referred to specialist arrhythmia clinics at Liverpool Heart and Chest Hospital for consideration of AF ablation were enrolled. All patients gave written informed consent for their procedures, and outcome data were extracted from an institutional review board‐approved registry. Obese patients attending one arrhythmia clinic were provided with the 5:2 dietary advice (diet group), and outcomes in these were compared to a propensity score matched (PSM) group of obese patients who received standard medical care with no specific dietary advice (control group). All other aspects of the management were identical between the two groups. The primary outcome was AF or atrial tachycardia (AT) recurrence on electrocardiographic monitoring within 12 months postablation. Secondary outcomes were periprocedural complications and change in body weight between clinic and ablation procedure.

### 2.1 Education and dietary advice

All patients in the diet group, along with their partners if available, were reviewed by an experienced consultant cardiologist (DG). Education was provided about the association between obesity and AF. They were counseled about the possibility that weight reduction could improve safety and efficacy of AF ablation, and were advised to follow the “Intermittent Fasting 5:2 diet”.^17^ This diet involves calorie restriction for 2 nonconsecutive days a week (less than 600 kcal/d for males, less than 500 kcal/d for females) with no restrictions on the remaining 5 days. Patients were requested to maintain a daily weight diary and to contact the hospital with their weight via telephone or email on a monthly basis. They were offered catheter ablation once they managed to lose at least 5 kg, or at 6 months even if not. This period was extended for patients who expressed a desire to lose more weight before coming in for their ablation.

Patients in the control group were assessed in two specialist arrhythmia clinics (RS and DG) where the management strategy was identical to those in the diet group, except that specialist dietary advice was not offered and weight loss was not specifically targeted.

### 2.2 Ablation procedure in both groups

Catheter ablation was performed either using point‐by‐point radiofrequency (RF) using CARTO or with a cryoballoon catheter. All RF pulmonary vein isolation (PVI) procedures were performed with wide‐area circumferential ablation using a Thermocool SmartTouch irrigated tip contact force‐sensing ablation catheter (Biosense Webster, Inc, California) through a deflectable sheath (Agilis NxT steerable introducer, Abbott, Inc, Minnesota). Ablations were delivered at least 10 mm outside the pulmonary vein (PV) ostia, in a power‐control mode with temperature limited to 48°C, maximal power output of 35‐40 W, and saline irrigation rate of 17 mL/min. Automated lesion tagging (VisiTag^TM^, Biosense Webster, Inc, California) was used for RF lesion marking, with a lesion display size of 3 mm. An intertag distance of ≤6 mm was aimed for, with target ablation index of >400 and ≥550 at the posterior and anterior walls, respectively. In cases performed under general anesthesia, esophageal temperature was monitored continuously, and RF delivery terminated if the esophageal temperature reached 38.5°C, or earlier at the operator's discretion. All patients were set out to have PVI‐only unless they had documented cavo‐tricuspid isthmus (CTI)‐dependent atrial flutter, in which case they also received CTI ablation. Patients with long‐standing persistent AF and dilated atria also underwent posterior wall isolation with creation of left atrial roof and floor lines at the operator's discretion. Prior to removal of the left atrial catheters, an assessment of each PV was undertaken. Any spontaneous PV reconnection was re‐isolated.

Cryoballoon ablation was performed using the 28 mm Arctic Front Advance cryoballoon (Medtronic Inc, Minneapolis, USA) and Achieve Advance Mapping catheter (Medtronic Inc, Minneapolis, USA). The aim was to attain a single 180 second “effective” freeze for each PV. A freeze is considered “effective” if either the time to PVI was less than 60s (if PV signals are discernible on any pole of the mapping catheter), or lowering of balloon temperature below −40°C at 60s (if PV signals not discernible on any pole of the mapping catheter).

Intravenous sheaths were removed following administration of intravenous protamine. Patients were mobilized after 4 hours bed rest, and discharged home the next day.

### 2.3 Follow‐up

Routine follow‐up visits were arranged at 3, 6, and 12 months. A 12‐lead electrocardiogram was recorded at each visit. Holter and other forms of ambulatory monitoring were performed for patients who had paroxysmal symptoms.

### 2.4 Statistical analyses

Continuous data were described with mean ± standard deviation (SD), and tested for differences using t‐test. Categorical data were described with counts and/or percentages, and tested for differences using Chi‐squared or Fisher's exact tests. Patients were matched based on propensity score generated by logistic regressions for age, sex, and AF type (paroxysmal/ nonparoxysmal) and preprocedural clinic BMI with a match tolerance of 0.1 and using the nearest‐neighbor technique without replacement. Incidence of AF/AT recurrence at 1 year was compared using logistic regression. The timing of AF/AT recurrence was contrasted using log‐rank test, and Kaplan‐Meier curves were generated. Subgroup analysis was performed within the diet group according to the amount of weight loss achieved prior to ablation: loss ≤10% of body weight (Group A) and loss >10% body weight (Group B). All tests were two‐sided, and a *P* value of <.05 was considered statistically significant. Analyses were performed using SPSS software version 24 (IBM Corp, Armonk, NY).

### 2.5 Patient and public involvement

Our patients were not involved in designing of, recruitment to, or conduct of the present study.

## 3 RESULTS

Fifty patients were provided with the 5:2 dietary advice while 92 received standard medical care. Following PSM, 92 patients (50 diet; 42 control) were included in the final analysis. Mean age was 64.6 (±9.1) years and 40 (44%) were females. Body weight and BMI at baseline were 105.0 (±15.3) kgs and 36.0 (±4.0), respectively. Fifty‐two (57%) patients had nonparoxysmal AF including 34 (37%) with long‐standing persistent AF. Baseline clinical characteristics were comparable between the two groups (Table 1).

**Table 1:** Baseline characteristics

	Diet (n = 50)	Control (n = 42)	*P* value
Age (y), mean ± SD	64.9 ± 9.5	64.1 ± 8.7	.66
Female sex	25 (50%)	15 (36%)	.17
BMI, mean ± SD	36.5 ± 4.0	35.3 ± 3.9	.16
Ischemic heart disease	5 (10%)	1 (2%)	.21
LA size (mm), mean ± SD	41.9 ± 5.2	41.7 ± 5.1	.88
Paroxysmal AF	19 (38%)	21 (50%)	.25
Medications			
Beta‐blockers	43 (86%)	35 (83%)	.72
Non‐dihydropyridine CCB	5 (10%)	2 (5%)	.45
AAD class I	11 (22%)	3 (7%)	.08
AAD class III	10 (20%)	7 (17%)	.68

Abbreviations: AAD, anti‐arrhythmic drug; AF, atrial fibrillation; BMI, body mass index; CCB, calcium‐channel blocker; LA, left atrial; SD, standard deviation.

Patients in the diet group achieved significantly more weight loss before their catheter ablation procedure compared to those in the control group (*P* < .01). Following a median of 5.3 (IQR 1.4‐15.6) months, the former group achieved a mean weight loss of 8.2 (±7.1) kgs, *P* < .01 compared to baseline, and their BMI at the time of the ablation procedure was 33.7 (±4.2), *P* < .01 compared to baseline. However, this was variable between patients; 36 (72%) patients achieved weight loss of less than 10% body weight (Group A) and 14 (28%) achieved weight loss greater than 10% body weight (Group B). About 37 (74%) patients achieved weight loss of greater than 5% body weight.

The ablation procedures were performed successfully in all 92 patients. The mean procedure duration was 152 (±94) minutes, and the mean ablation time was 31 (±15) minutes. Seventy (76%) patients underwent RF ablation while 22 (24%) underwent cryoballoon PVI. More patients underwent posterior wall isolation in the diet group (44%) compared to controls (19%), *P* = .011. One procedural complication (1.1%) occurred in the entire cohort. This patient from the diet group developed postablation pulmonary edema that required management on intensive care overnight, and delayed hospital discharge by 2 days.

**Figure 1:**
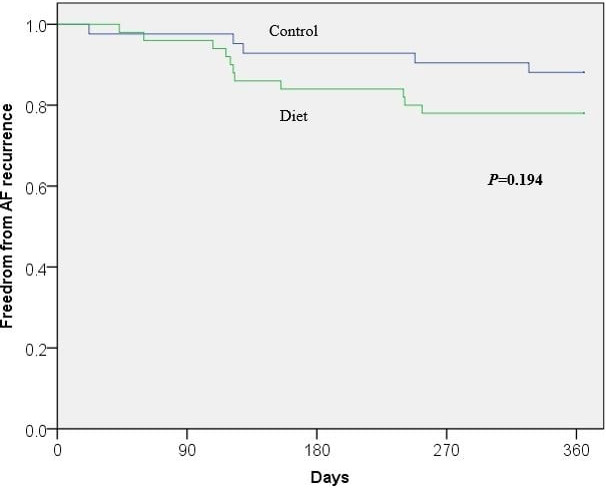
Kaplan‐Meier analysis of freedom from AF recurrence in diet vs control group

Overall, 16 (17%) patients had AF/AT recurrence at 1 year. The rate of AF/AT recurrence in this period was not significantly different between the groups, 22% diet vs 12% control, *P* = .21 (Table 2). The mean follow‐up duration for the diet group was 23.3 (±11.4) months and for the control group was 24.3 (±13.5) months, *P* = .69. Using Kaplan‐Meier analysis, the freedom from AF/AT was similar in both groups, log‐rank *P* = .13 (Figure 1).

**Table 2:** Efficacy of AF ablation at 1 year in control vs diet group

	Diet (n = 50)	Control (n = 42)	*P* value
AF recurrence	11 (22%)	5 (12%)	.21
Symptom frequency			
Improved	7 (64%)	4 (80%)	
Unchanged	4 (36%)	1 (20%)	
Symptom duration			
Improved	6 (55%)	4 (80%)	
Unchanged	5 (46%)	1 (20%)	
Further cardioversion	7 (64%)	2 (40%)	

Abbreviation: AF, atrial fibrillation.

### 3.1 Outcomes according to extent of weight loss

**Figure 2:**
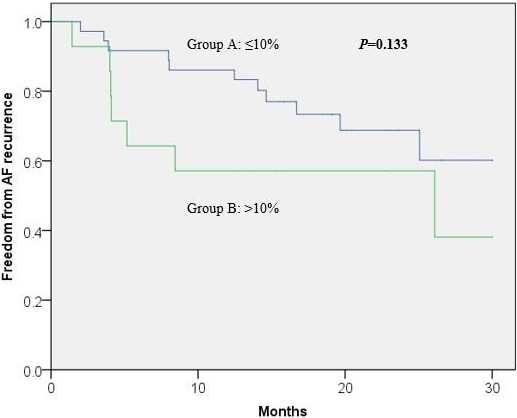
Kaplan‐Meier analysis of freedom from AF recurrence based on weight reduction in the diet group

Body weight at the time of ablation was 100.9 (±16.8) kgs and 96.3 (±14.8) kgs, and BMI was 34.3 (±4.2) and 32.4 (±3.9) for Groups A and B, respectively. Only four (8%) patients had World Health Organization Class III obesity (BMI ≥ 40) at the time of ablation as compared to 11 (22%) patients at time of initial assessment, *P* = .03. The recurrence of AF/AT at 12 months was significantly higher in Group B (42.9% vs 13.9%), *P* = .04. However, Kaplan‐Meier analysis demonstrated no statistical difference in AF/AT recurrence between the groups over a 30 month follow‐up period, log‐rank *P* = .13 (Figure 2).

### 3.2 Outcomes according to BMI at procedure

**Figure 3:**
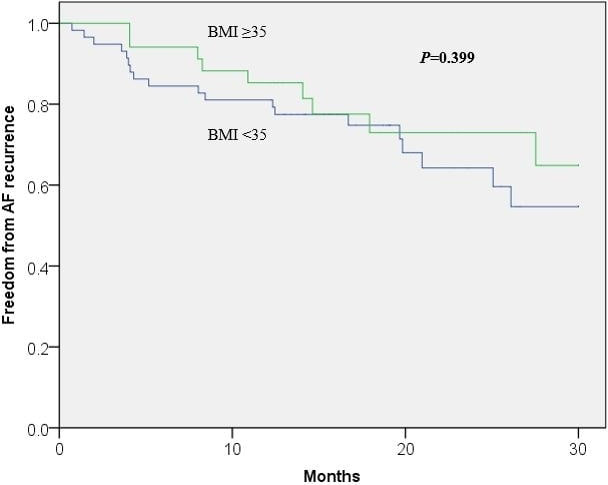
Kaplan‐Meier analysis of freedom from AF recurrence based on BMI at the time of ablation

We performed further analyses according to patients’ BMI at the time of procedure. Fifty‐eight (63%) patients had a BMI < 35 and 34 (37%) patients had a BMI ≥ 35. There was no difference in outcomes of AF/AT recurrence at 1 year between these groups, 15% (BMI ≥ 35) vs 19% (BMI < 35), *P* = .60 (Table 3). Kaplan‐Meier analysis demonstrated similar results over a 30‐month follow‐up period, log‐rank *P* = .40 (Figure 3).

**Table 3:** Efficacy of AF ablation at 1 year according to BMI at the time of ablation

	BMI < 35 (n = 58)	BMI ≥ 35 (n = 34)	*P* value
BMI, mean ± SD	31.9 ± 1.8	38.8 ± 3.1	<.01
AF recurrence	11 (19%)	5 (15%)	.60
Symptom frequency			
Improved	7 (64%)	4 (80%)	
Unchanged	4 (36%)	1 (20%)	
Symptom duration			
Improved	6 (55%)	4 (80%)	
Unchanged	5 (46%)	1 (20%)	
Further cardioversion	6 (55%)	3 (60%)	

Abbreviations: AF, atrial fibrillation; BMI, body mass index; SD, standard deviation.

## 4 DISCUSSION

This “real‐world” study demonstrates that (a) it is possible to achieve significant weight reduction in obese AF patients in a nonbariatric arrhythmia clinic with simple education and dietary advice, (b) modern AF ablation has excellent procedural safety as well as medium‐term arrhythmia‐free survival rates in this traditionally difficult population of obese patients with a high proportion of nonparoxysmal AF, and (c) significant weight reduction, even beyond the proposed threshold of over 10% body weight, is achievable but in isolation does not appear to translate to better arrhythmia outcomes.

Physicians are sometimes reluctant to consider AF ablation in obese patients because of the perceived higher complication risks and poor success rates.^11^, ^12^, ^13^, ^14^ As such, health systems are reviewing the cost efficacy of AF ablation in this cohort. In fact, NHS England has recently released a draft of their proposed commissioning guidelines according to which AF ablation will not be offered to patients with BMI greater than 40, and patients with BMI between 35 and 40 will need to demonstrate weight reduction of at least 10% of their body weight before being eligible for AF ablation. The feasibility of this degree of weight reduction in patients referred to a stand‐alone specialist arrhythmia clinic with no support from bariatric or dietetic services has never been studied. Furthermore, with the recent advances in AF ablation techniques and protocols, its efficacy and safety in this cohort of patients need to be re‐evaluated in a contemporary series. We have seen in our study that the major complication rates were low. This is almost certainly because the most common complications of AF ablation have historically been related to vascular access, and these are particularly more likely in obese patients.^18^, ^19^ Since the introduction of ultrasound to obtain vascular access, these complications have been all but eradicated.^20^ The risk of cardiac tamponade has been mitigated in recent years by the use of transesophageal echocardiography to guide transseptal punctures, and the introduction of contact force‐sensing and cryoballoon ablation catheters, both of which were used in this study.

Recently, several studies from a single center have been published demonstrating the benefits of weight loss in patients with AF.^15^, ^21^, ^22^, ^23^ Of these, the ARREST‐AF trial was designed to evaluate outcomes specifically in patients undergoing AF ablation. It is important to note that these studies employed a multifactorial risk factor modification approach that included blood pressure control, lipid management, glycemic control, sleep‐disordered breathing management, and smoking cessation and alcohol reduction support.^15^ On the other hand, in a study focused on weight reduction alone, Mohanty et al showed no correlation between weight loss and success of ablation in patients with long‐standing persistent AF.^16^ As such, the role of weight loss on the success of catheter AF ablation remains poorly established.

Our findings are consistent with those by Mohanty *et al* Furthermore, BMI at the time of procedure was not associated with outcomes of AF ablation in our obese cohort. This may indicate that certain features of cardiac remodeling secondary to obesity^24^ may be irreversible. Indeed, some studies have shown that despite improvement in left ventricular parameters with weight loss in obese patients, the left atrial size remained fixed.^25^, ^26^, ^27^ Consequently, similar to structural and electrical remodeling due to AF, irreversible changes may occur once a certain obesity threshold is exceeded. In addition, any possible benefits of weight loss need to be balanced against the significant time taken for weight loss measures to take effect. It is well described that increasing duration of AF has negative effects on ablation success rates. In our study, the median time from clinic to ablation procedure among patients who achieved more than 10% weight loss in the diet group was 10 months, and this delay may have contributed to the observation of higher AF recurrence. Nonetheless, it is important to recognize that though weight loss in obese patients may not reduce AF recurrence postablation, it may have several other important health benefits and we continue to believe that it should be encouraged among these patients.

Weight loss can be challenging for obese patients who are symptomatic from AF. In the ARREST‐AF trial, this was achieved through a dedicated physician‐directed weight management clinic.^15^ Participants were provided with motivational and goal‐directed counseling, a meal plan, behavior modification, a diet and physical activity diary, and a meal replacement as required. Despite evidence to support the cost‐effectiveness of this strategy in one specialized medical center in Australia,^28^ it may not be applicable globally to the general medical and cardiology clinics, where the majority of patients with AF are managed. Indeed, to the best of our knowledge, those impressive results have not yet been replicated by any other center. Therefore, it is important to identify simple and practical tools that may be used to encourage weight loss in these situations. To this end, we have shown that simple dietary advice of intermittent fasting 5:2 diet may be effective in promoting weight loss in obese patients with AF, whereby three‐quarters of patients were able to lose at least 5% of their baseline body weight prior to AF ablation. Furthermore, it may be incorporated into their usual clinic review without the need to commit additional resources.

### 4.1 Limitations

The main limitation of this study is its retrospective observational study design and small sample size which may lead to variability in the results obtained. These factors together with a greater proportion (though not statistically significant) of patients with nonparoxysmal AF in those who had >10% weight loss in the diet group may have contributed to the counter‐intuitive results of higher AF recurrence in this group of patients. However, some of the weaknesses typically associated with an observational study design were addressed using PSM. A second limitation is that we were unable to confirm whether the weight loss was sustained in a significant proportion of patients. The importance of sustained weight loss postablation was previously demonstrated to be associated with a lower AF recurrence rate.^29^ Among the 22 (44%) in the diet group, where these data are available, the weight loss achieved preablation was maintained during follow‐up in all. Thirdly, the success rates described in this study are “pragmatic” success rates, as evaluated from a patient's clinical perspective with limited electrocardiographic monitoring. It is likely that some instances of asymptomatic paroxysmal AF would have been missed. The post hoc analysis of patients according to BMI subgroups includes a heterogeneous cohort of patients who received dietary advice and controls, and therefore should be interpreted with caution.

## 5 CONCLUSIONS

We show that significant weight reduction in obese AF patients is achievable in a tertiary arrhythmia clinic with simple dietary advice. Excellent outcomes of catheter AF ablation are seen in this historically difficult cohort with modern ablation techniques. Significant weight loss alone does not translate to better outcomes; whether this is because of advanced arrhythmia substrate by the time patients are referred to an arrhythmia clinic, or because of the associated delay in ablation caused by the efforts at further weight reduction needs to be evaluated in further studies.

## CONFLICT OF INTEREST

WYD, SHC, MS, and RS: None declared. NK: Grants from the Swiss National Science Foundation (P400PM‐194477), Gottfried und Julia Bangerter‐Rhyner‐Stiftung, and the European Society of Cardiology. GYHL: Consultant for Bayer/Janssen, BMS/Pfizer, Medtronic, Boehringer Ingelheim, Novartis, Verseon and Daiichi‐Sankyo. Speaker for Bayer, BMS/Pfizer, Medtronic, Boehringer Ingelheim, and Daiichi‐Sankyo. No fees are directly received personally. DG: Speaker for Bayer, BMS/Pfizer, Boehringer Ingelheim, Daiichi‐Sankyo, Medtronic, Biosense Webster and Boston Scientific. Proctor for Abbott. Research Grants from Medtronic, Biosense Webster and Boston Scientific.

## References

[1] Chugh SS , Havmoeller R , Narayanan K , Singh D , Rienstra M , Benjamin EJ , et al. Worldwide epidemiology of atrial fibrillation: a Global Burden of Disease 2010 Study. Circulation. 2014;129(8):837–47. 24345399 10.1161/CIRCULATIONAHA.113.005119 PMC4151302

[2] Stewart S , Hart CL , Hole DJ , McMurray JJ . A population‐based study of the long‐term risks associated with atrial fibrillation: 20‐year follow‐up of the Renfrew/Paisley study. Am J Med. 2002;113(5):359–64. 12401529 10.1016/s0002-9343(02)01236-6

[3] Thrall G , Lane D , Carroll D , Lip GYH . Quality of life in patients with atrial fibrillation: a systematic review. Am J Med. 2006;119(5):448.e1–19. 10.1016/j.amjmed.2005.10.057 16651058

[4] Vermond RA , Geelhoed B , Verweij N , Tieleman RG , Van der Harst P , Hillege HL , et al. Incidence of atrial fibrillation and relationship with cardiovascular events, heart failure, and mortality A community‐based study from the Netherlands. J Am Coll Cardiol. 2015;66(9):1000–7. 26314526 10.1016/j.jacc.2015.06.1314

[5] Goudis CA , Korantzopoulos P , Ntalas IV , Kallergis EM , Ketikoglou DG . Obesity and atrial fibrillation: a comprehensive review of the pathophysiological mechanisms and links. J Cardiol. 2015;66(5):361–9. 25959929 10.1016/j.jjcc.2015.04.002

[6] Kloosterman M , Oldgren J , Conen D , Wong JA , Connolly SJ , Avezum A , et al. Characteristics and outcomes of atrial fibrillation in patients without traditional risk factors: an RE‐LY AF registry analysis. Europace. 2020. 10.1093/europace/euz360 PMC7273333 32215649

[7] Wang TJ , Parise H , Levy D , D’Agostino RBS , Wolf PA , Vasan RS , et al. Obesity and the risk of new‐onset atrial fibrillation. JAMA. 2004;292(20):2471–7. 15562125 10.1001/jama.292.20.2471

[8] Al‐Omari MA , Finstuen J , Appleton CP , Barnes ME , Tsang TSM . Echocardiographic assessment of left ventricular diastolic function and filling pressure in atrial fibrillation. Am J Cardiol. 2008;101(12):1759–65. 18549855 10.1016/j.amjcard.2008.02.067

[9] Lioni L , Korantzopoulos P , Letsas KP . Catheter ablation of atrial fibrillation in overweight and obese patients. J Atr Fibrillation. 2011;4(4):1216. 28496711 10.4022/jafib.454 PMC5153096

[10] Chalazan B , Dickerman D , Sridhar A , Farrell M , Gayle K , Samuels DC , et al. Relation of body mass index to symptom burden in patients with atrial fibrillation. Am J Cardiol. 2018;122(2):235–41. 29914646 10.1016/j.amjcard.2018.04.011 PMC6028292

[11] Wong CX , Sullivan T , Sun MT , Mahajan R , Pathak RK , Middeldorp M , et al. Obesity and the risk of incident, post‐operative, and post‐ablation atrial fibrillation: a meta‐analysis of 626,603 individuals in 51 studies. JACC Clin Electrophysiol. 2015;1(3):139–52. 29759357 10.1016/j.jacep.2015.04.004

[12] Guijian L , Jinchuan Y , Rongzeng D , Jun Q , Jun W , Wenqing Z . Impact of body mass index on atrial fibrillation recurrence: a meta‐analysis of observational studies. Pacing Clin Electrophysiol. 2013;36(6):748–56. 23437987 10.1111/pace.12106

[13] Cha Y‐M , Friedman PA , Asirvatham SJ , Shen W‐K , Munger TM , Rea RF , et al. Catheter ablation for atrial fibrillation in patients with obesity. Circulation. 2008;117(20):2583–90. 18474813 10.1161/CIRCULATIONAHA.107.716712

[14] Letsas KP , Siklody CH , Korantzopoulos P , Weber R , Burkle G , Mihas CC , et al. The impact of body mass index on the efficacy and safety of catheter ablation of atrial fibrillation. Int J Cardiol. 2013;164(1):94–8. 21726910 10.1016/j.ijcard.2011.06.092

[15] Pathak RK , Middeldorp ME , Lau DH , Mehta AB , Mahajan R , Twomey D , et al. Aggressive risk factor reduction study for atrial fibrillation and implications for the outcome of ablation: the ARREST‐AF cohort study. J Am Coll Cardiol. 2014;64(21):2222–31. 25456757 10.1016/j.jacc.2014.09.028

[16] Mohanty S , Mohanty P , Natale V , Trivedi C , Gianni C , Burkhardt JD , et al. Impact of weight loss on ablation outcome in obese patients with longstanding persistent atrial fibrillation. J Cardiovasc Electrophysiol. 2018;29(2):246–53. 29171116 10.1111/jce.13394

[17] Scholtens EL , Krebs JD , Corley BT , Hall RM . Intermittent fasting 5:2 diet: What is the macronutrient and micronutrient intake and composition? Clin Nutr. 2020. 10.1016/j.clnu.2020.02.022 32199696

[18] Shoemaker MB , Muhammad R , Farrell M , Parvez B , White BW , Streur M , et al. Relation of morbid obesity and female gender to risk of procedural complications in patients undergoing atrial fibrillation ablation. Am J Cardiol. 2013;111(3):368–73. 23168290 10.1016/j.amjcard.2012.10.013 PMC3546280

[19] Winkle RA , Mead RH , Engel G , Kong MH , Fleming W , Salcedo J , et al. Impact of obesity on atrial fibrillation ablation: patient characteristics, long‐term outcomes, and complications. Heart Rhythm. 2017;14(6):819–27. 28232261 10.1016/j.hrthm.2017.02.023

[20] Stroker E , de Asmundis C , Kupics K , Takarada K , Mugnai G , De Cocker J , et al. Value of ultrasound for access guidance and detection of subclinical vascular complications in the setting of atrial fibrillation cryoballoon ablation. Europace. 2019;21(3):434–9. 30010776 10.1093/europace/euy154

[21] Pathak RK , Elliott A , Middeldorp ME , Meredith M , Mehta AB , Mahajan R , et al. Impact of cardiorespiratory fitness on arrhythmia recurrence in obese individuals with atrial fibrillation: the CARDIO‐FIT Study. J Am Coll Cardiol. 2015;66(9):985–96. 26113406 10.1016/j.jacc.2015.06.488

[22] Pathak RK , Middeldorp ME , Meredith M , Mehta AB , Mahajan R , Wong CX , et al. Long‐term effect of goal‐directed weight management in an atrial fibrillation cohort: a long‐term follow‐up study (LEGACY). J Am Coll Cardiol. 2015;65(20):2159–69. 25792361 10.1016/j.jacc.2015.03.002

[23] Abed HS , Wittert GA , Leong DP , Shirazi MG , Bahrami B , Middeldorp ME , et al. Effect of weight reduction and cardiometabolic risk factor management on symptom burden and severity in patients with atrial fibrillation: a randomized clinical trial. JAMA. 2013;310(19):2050–60. 24240932 10.1001/jama.2013.280521

[24] Abel ED , Litwin SE , Sweeney G . Cardiac remodeling in obesity. Physiol Rev. 2008;88(2):389–419. 18391168 10.1152/physrev.00017.2007 PMC2915933

[25] Garza CA , Pellikka PA , Somers VK , Sarr MG , Collazo‐Clavell ML , Korenfeld Y , et al. Structural and functional changes in left and right ventricles after major weight loss following bariatric surgery for morbid obesity. Am J Cardiol. 2010;105(4):550–6. 20152253 10.1016/j.amjcard.2009.09.057

[26] Garza CA , Pellikka PA , Somers VK , Sarr MG , Seward JB , Collazo‐Clavell ML , et al. Major weight loss prevents long‐term left atrial enlargement in patients with morbid and extreme obesity. Eur J Echocardiogr. 2008;9(5):587–93. 18490311 10.1093/ejechocard/jen117 PMC2724883

[27] Shin S‐H , Lee YJ , Heo Y‐S , Park S‐D , Kwon S‐W , Woo S‐I , et al. Beneficial effects of bariatric surgery on cardiac structure and function in obesity. Obes Surg. 2017;27(3):620–5. 27510590 10.1007/s11695-016-2330-x

[28] Pathak RK , Evans M , Middeldorp ME , Mahajan R , Mehta AB , Meredith M , et al. Cost‐effectiveness and clinical effectiveness of the risk factor management clinic in atrial fibrillation: The CENT Study. JACC Clin Electrophysiol. 2017;3(5):436–47. 29759599 10.1016/j.jacep.2016.12.015

[29] Jia ZX , Jiang C , Lu SX , Liu JP , Guo XY , Li SN , et al. Association between weight control and recurrence of atrial fibrillation after catheter ablation in overweight and obese patients. Zhonghua Xin Xue Guan Bing Za Zhi. 2019;47(8):595–601. 31434429 10.3760/cma.j.issn.0253-3758.2019.08.002

